# 124 Mixed Signals: The Ethics of Caring for Burn Patients Whose Goals and Choices Diverge

**DOI:** 10.1093/jbcr/iraf019.124

**Published:** 2025-04-01

**Authors:** Anna Goff, Andrew Greenway, Barrie Huberman

**Affiliations:** Weill Cornell Medicine; New York Presbyterian Hospital; Weill Cornell Medicine

## Abstract

**Introduction:**

Surviving severe burns requires repeated exposure to painful therapeutic interventions. As a result, a patient’s ability and willingness to tolerate treatment can directly impact their prognosis. In this project, we consider the ethical implications of patients who want to survive yet have a low tolerance for experiencing treatment due to pain, anxiety, or a desire for control that manifests in acute refusals of care.

**Methods:**

We first conducted a literature review on treatment refusals in burn care. We then engaged in a normative analysis of ethical considerations in cases where patients’ acute treatment refusals are value-discordant with their own survival-oriented goals.

**Results:**

Dax Cowart’s story has long-dominated discourse on treatment refusals in burn care. Severely burned in 1973 and treated over his objection, Cowart spent the rest of his life arguing that providers ought to have heeded his refusals and respected his wish to die. Cowart’s case and others described in the literature reflect a fundamental tension between the ethical principles of respect for autonomy and beneficence. While important, cases such as Cowart’s are not illustrative of all situations in which burn providers encounter treatment refusals. Rather, the nature of burn care is such that providers often face the difficult question of whether, and when, to treat a patient over their objection in pursuit of their broader desire to live.

One challenge facing providers is that repeated refusals tend to have aggregate effects on patient recovery. For example, where missing one therapy session might be non-ideal, missing a week could have significant negative consequences. Similarly, refusing to be turned once might be insignificant, but not allowing turns for multiple hours poses a greater risk. Burn providers often find themselves having to balance their obligation to respect patient wishes, including both their desire for treatment and their refusals, with their duty promote a patient’s recovery and well-being, which often involves continued engagement in care. This conflict pose challenges to providing goal-oriented care, raises difficult questions regarding the bounds of pain management and palliative sedation, and puts burn providers at risk of experiencing moral distress and burnout.

**Conclusions:**

While compelling, Cowart’s case is largely concerned with treatment refusal in the global sense, insofar as his refusals were consistent with an overarching wish to die. Our work demonstrates the ethically nuanced and challenging nature of burn patient refusals, especially when those refusals are inconsistent with a patient’s stated treatment goals.

**Applicability of Research to Practice:**

This presentation broadens the discussion of treatment refusals in burn care beyond Cowart’s case and highlights the importance of open communication about conflicting ethical duties in patient care.

**Funding for the Study:**

N/A

